# Two-dimensional semiconductors ZrNCl and HfNCl: Stability, electric transport, and thermoelectric properties

**DOI:** 10.1038/s41598-017-17590-w

**Published:** 2017-12-11

**Authors:** Won Seok Yun, J. D. Lee

**Affiliations:** Department of Emerging Materials Science, DGIST, Daegu, 42988 Republic of Korea

## Abstract

Searching for novel two-dimensional (2D) semiconducting materials is a challenging issue. We investigate novel 2D semiconductors ZrNCl and HfNCl which would be isolated to single layers from van der Waals layered bulk materials, i.e., ternary transition-metal nitride halides. Their isolations are unquestionably supported through an investigation of their cleavage energies as well as their thermodynamic stability based on the *ab initio* molecular dynamics and phonon dispersion calculations. Strain engineering is found to be available for both single-layer (1L) ZrNCl and 1L-HfNCl, where a transition from an indirect to direct band gap is attained under a tensile strain. It is also found that 1L-ZrNCl has an excellent electron mobility of about 1.2 × 10^3^ cm^2^ V^−1^ s^−1^, which is significantly higher than that of 1L-MoS_2_. Lastly, it is indicated that these systems have good thermoelectric properties, i.e., high Seebeck coefficient and high power factor. With these findings, 1L-ZrNCl and 1L-HfNCl would be novel promising 2D materials for a wide range of optoelectronic and thermoelectric applications.

## Introduction

Recently, due to the development of the exfoliation technique, it has been possible to obtain atomic-layered sheets from various layered bulk materials^1,2^. Thereafter, 2D materials have gained much interest due to their potential applications in areas, such as electronics, optoelectronics, photovoltaics, lithium ion batteries, spintronic devices, and so on^1–7^. Among such 2D layered materials, in particular, transition-metal dichalcogenides (TMDC) have attained the utmost attention and been extensively studied so far due to their intriguing electronic and optical properties^8–10^. Black phosphorous also attracts a great attention because it shows high carrier mobility at room temperature and also preserves a direct band gap regardless of the number of layers^11^. Likewise, due to a huge potential to upstage the electronics and spintronics, it is a central issue to search for novel 2D materials with novel functionalities.

Ternary transition-metal nitride halides (TMNH) contains two types of layer-structured polymorphs; the *α*- and *β*-formed isomorphous with orthogonal FeOCl-type and hexagonal SmSI-type layered structures, respectively^12–14^. Both are known to be changed to superconductors with moderately high transition temperatures up to 25.5 K upon electron-doping by means of intercalation through the interlayer space^15,16^. During the last few decades, as a matter of fact, ternary TMNH have been intensively studied mainly for their superconductivity^14–18^. Hence, not only their 2D structures but also the realization possibilities have received little attention. Nevertheless, a single-layer (1L) TMNH has been recently suggested simply from the fact that the layers of TMNH are bonded by van der Waals (vdW) interaction like graphite and TMDC^19^. It is composed of double transition-metal (group IV elements *M*; Ti, Zr, and Hf) nitride layers (*M*-N-N-*M*) sandwiched by halogen (Cl, Br, and I) layers. However, scientific details of 1L-TMNH including its stability are absolutely lacking.

A suggestion of 1L-TMNH is followed by several natural questions. First, it should be scrutinized whether the stable 1L-TMNH can be actually exfoliated from the bulk TMNH. That is, the exfoliation possibility as well as the thermodynamic stability of 1L-TMNH should be examined. Second, it is questionable whether 1L-TMNH could exhibit better electronic and mechanical properties compared to the bulk TMNH. The 2D materials currently under a lot of consideration show such superb merits in tuning the electronic structure like the gap modulating and strain engineering as the parent bulk material does not have. Third, some of 2D materials undergo a substantial change in the transport properties, as compared to the bulk. It should be intriguing to investigate the electric or thermoelectric transport properties of 1L-TMNH.

In this paper, performing the first-principles calculations to examine extensively the stability, electronic structure, electric transport, and thermoelectric properties, we introduce novel promising 2D semiconductors 1L-ZrNCl and 1L-HfNCl. Both of them are shown to be easily isolated from the parent bulk materials and also thermodynamically stable. In addition, it is found that both systems show a transition from an indirect to direct band gap under the tensile strain, which is important for optoelectronic device applications. Especially, 1L-ZrNCl has an outstanding electron mobility of about 1.2 × 10^3^ cm^2^ V^−1^ s^−1^ in the *a*- or *b*-direction. Such electron mobility is significantly higher than that of 1L-MoS_2_, whereas the hole mobility is about two orders of magnitude lower. Furthermore, due to their high Seebeck coefficient and high power factor, these systems are found to be good candidates for promising thermoelectric materials.

## Computational Details

The first-principles calculations were performed using density functional theory (DFT) as implemented in the Vienna *ab initio* simulation package (VASP) code^20^. The electron-ion interactions were described by the projector augmented wave (PAW) method^21,22^ and the exchange-correlation potential was expressed using Perdew-Burke-Ernzerhof (PBE) generalized gradient approximation (GGA) functional^23^. An energy cutoff of 500 eV was adopted for the plane-wave expansion of the electronic wave function and a 24 × 24 × 1 Monkhorst-Pack **k**-point grid was used for an integration over the 2D Brillouin zone. The present work did not consider the spin-orbit coupling (SOC) effect because the results of this study were negligibly affected by that effect (Fig. S1). The unit cell was optimized to obtain the equilibrium lattice parameter at the lowest total energy and atomic positions were fully relaxed until the force on each atom was less than 10^−4^ eVÅ^−1^. The vacuum space along the *z* direction was taken to be more than 15 Å for all the considered systems and the convergence criterion in the self-consistency process was set to 10^−4^ eV. In order to consider interlayer vdW interactions, the optimized exchange vdW functional (optB88-vdW)^24,25^ was employed. The phonon spectra calculations were performed using the finite displacement approach, as implemented in the Phonopy code^26^, in which a 3 × 3 × 1 supercell was employed.

To examine the thermodynamic stability of 1L-ZrNCl and 1L-HfNCl at room temperature, *ab initio* molecular dynamics (AIMD) simulations were performed at 300 K within the framework of DFT. In the AIMD calculations, the canonical ensemble with a Nosé-Hoover thermostat for temperature control was used^27^. The simulation was performed for 10 ps with a time step of 1 fs. The Γ point alone was used to sample the Brillouin zone of the 3 × 3 × 1 supercell. The carrier mobility was obtained with the deformation potential (DP) theory proposed by Bardeen and Shockley^28^ because the coherent wavelength of thermally activated electrons or holes at room temperature is close to the acoustic phonon wavelength and much larger than the lattice constant. The analytical expression of the acoustic-phonon-limited carrier mobility (*μ*) in 2D materials under the effective mass approximation is as follows:

1 $${\mu }_{{\rm{2D}}}=\frac{2e{\hslash }^{3}{C}_{{\rm{2D}}}}{3{E}_{1}^{2}{k}_{B}T|{m}^{\ast }{|}^{2}},$$

where m* is the carrier effective mass calculated by ${m}^{\ast }={\hslash }^{2}{[{\partial }^{2}\varepsilon (\overrightarrow{k})/\partial {k}^{2}]}^{-1}$ and *T* is the temperature. In the 2D case, the stretching modulus (*C*_2D_) is defined as *C*_2D_ = (∂^2^ *E*/∂*δ* ^2^)/*S*_0_, where *E* and *S*_0_ are the total energy and area of supercell, respectively, and *δ* = Δ*l*/*l*_0_, where Δ*l* is the deformation of the lattice constant by the uniaxial strain and *l*_0_ is the value at equilibrium geometry. Moreover, *E*_1_ is the DP constant defined as *E*_1_ = Δ*E*_edge_/Δ*δ*, where Δ*E*_edge_ is the energy shift of the band edge of the valence band or conduction band with respect to lattice dilation along the direction of external strain. This DP method has been successfully applied to predict the mobility of 2D materials, such as graphene^29^, MoS_2_^30,31^, and phosphorene^11^.

For the thermoelectric transport properties, we adopted the semi-classical Boltzmann transport theory as incorporated in the BoltzTraP code^32^ within the constant relaxation time approximation and rigid band approximation. Based on this approximation, the electrical conductivity and Seebeck coefficient tensors of a material can be written as^32^

2 $${\sigma }_{\alpha \beta }(T,\mu )=\frac{1}{{\rm{\Omega }}}\,\int \,{\sigma }_{\alpha \beta }(\varepsilon )[-\frac{\partial {f}_{\mu }(T,\varepsilon )}{\partial \varepsilon }]\,d\varepsilon $$

3 $${S}_{\alpha \beta }(T,\mu )=\frac{1}{{\sigma }_{\alpha \beta }(T,\mu )eT{\rm{\Omega }}}\,\int \,{\sigma }_{\alpha \beta }(\varepsilon )\,(\varepsilon -\mu )\,[-\frac{\partial {f}_{\mu }(T,\varepsilon )}{\partial \varepsilon }]\,d\varepsilon $$

where *α* and *β* are tensor indices and *μ*, Ω, and *e* are the chemical potential, the volume of the unit cell, and the electron charge, respectively. Subsequently, *f*_*μ*_ and *σ*_*αβ*_(*ε*) are the Fermi-Dirac distribution function and the energy-projected conductivity tensor, respectively. Moreover, we used a 50 × 50 × 1 **k**-mesh to obtain the Seebeck coefficient *S*, electrical conductivity *σ*, and power factor (PF) *S* ^2^ *σ*.

## Results and Discussion

### Crystal structures and exfoliation possibilities

**Figure 1:**
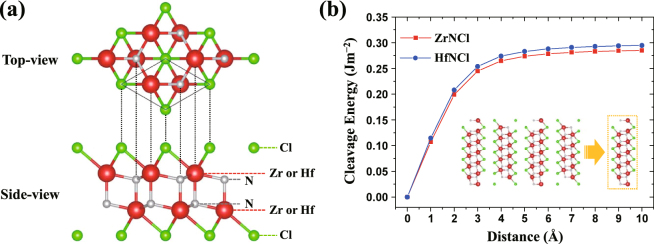
(**a**) Top- and side-views of the crystal structure of 1L-ZrNCl (or 1L-HfNCl). (**b**) Cleavage energy as a function of the separation distance between 1L and the quadruple-layer (4L). The inset shows a schematic illustration of the considered exfoliation.

The top and side-views of the atomic structure of the 1L-ZrNCl (or 1L-HfNCl) with the *β*-form are shown in Fig. 1(a). As shown in Fig. 1(a), the *β*-form 1L-ZrNCl (or 1L-HfNCl) is composed of a honeycomb-like double Zr(Hf)–N layer sandwiched between the halogen Cl layers. Note that 1L-ZrNCl is calculated to be more stable in the *β*-form than in the *α*-form by about 127 meV/unit-cell. Therefore, we hereafter focus on *β*-1L-ZrNCl (also *β*-1L-HfNCl), unless mentioned otherwise. The optimized lattice constants of the 1L-ZrNCl and 1L-HfNCl were calculated to be 3.631 and 3.591 Å, respectively, in good agreement with a previous theoretical study^19^.

To assess the possibility of obtaining 1L-ZrNCl (or 1L-HfNCl) from its bulk crystal by mechanical exfoliation techniques, here we compute the cleavage energy. The cleavage energy (*E*_cl_) of ZrNCl (or HfNCl) is defined as follows:

4 $${E}_{{\rm{cl}}}={E}_{{\rm{1L}}}+{E}_{{\rm{5L}}-{\rm{1L}}}^{{\rm{after}}}-{E}_{{\rm{5L}}}^{{\rm{before}}},$$

where *E*_1L_, ${E}_{{\rm{5L}}-{\rm{1L}}}^{{\rm{after}}}$, and ${E}_{{\rm{5L}}}^{{\rm{before}}}$ are the total energy of 1L-ZrNCl (or 1L-HfNCl), the total energy of the quintuple-layer (5L) system after exfoliation, and the total energy of the 5L system before exfoliation, respectively. Note that 5L slab is regarded as a model of the bulk. The resulting cleavage energies of ZrNCl and HfNCl as a function of the separation distance are shown in Fig. 1(b). As the separation distance increases, the cleavage energies gradually increase and are eventually saturated to ~0.29 Jm^−2^ and ~0.30 Jm^−2^ for the ZrNCl and HfNCl sheets, respectively. Interestingly, these values are lower than the experimentally determined cleavage energy of 0.36 Jm^−2^ of graphite^33^. This implies that it possibly warrants achieving 1L-ZrNCl (or 1L-HfNCl) from their bulk crystal experimentally. Furthermore, this also indicates that those materials have a relatively weak vdW interaction.

### Thermodynamic stabilities

**Figure 2:**
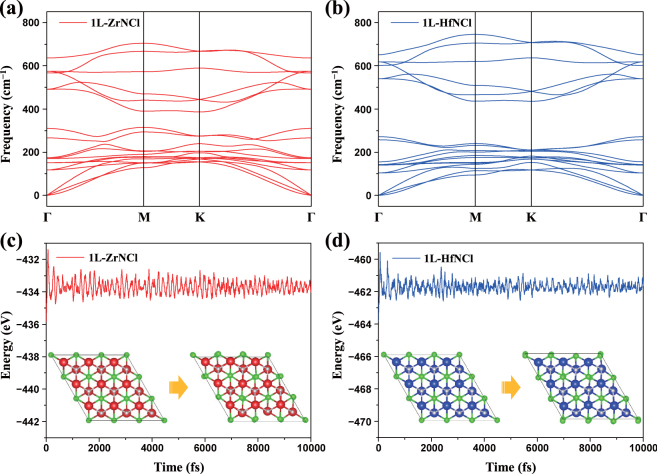
Phonon dispersion of (**a**) 1L-ZrNCl and (**b**) 1L-HfNCl. Total energy fluctuation during the AIMD simulation for (**c**) 1L-ZrNCl and (**d**) 1L-HfNCl at 300 K. The insets in (**c**,**d**) show the snapshots of the atomic configuration at 0 ps (left) and 10 ps (right).

To confirm the thermodynamic stability, the phonon dispersion curves along the high-symmetry lines of the first Brillouin zone are presented in Fig. 2(a,b) for 1L-ZrNCl and 1L-HfNCl, respectively. There is found no appreciable negative frequency in the phonon spectra, saying that both 1L-ZrNCl and 1L-HfNCl are dynamically stable. Moreover, the highest frequencies of 1L-ZrNCl and 1L-HfNCl reach up to 21.11 THz (704.15 cm^−1^) and 22.36 THz (745.85 cm^−1^), respectively. It should be noted that these values are higher than the highest frequencies of 1L-MoS_2_ (473 cm^−1^) and 1L-WS_2_ (442 cm^−1^)^34^. These high-frequency phonons indicate the robust Zr-N (Hf-N) and Zr-Cl (Hf-Cl) bondings in 1L-ZrNCl (1L-HfNCl). Subsequently, *ab initio* molecular dynamics (AIMD) simulations were done at 300 K for 1L-ZrNCl and 1L-HfNCl. As shown in the snapshots of Fig. 2(c,d), the in-plane structures are well maintained after 10 ps, suggesting good thermodynamic stabilities of the 1L-ZrNCl and 1L-HfNCl sheets. In addition, even though the energy fluctuations at high temperatures (500 K and 800 K) become larger than those at room temperature as shown in Fig. S2, there are found neither structure disruption nor structure reconstruction in the systems still after 10 ps. Therefore, we conclude that 1L-ZrNCl and 1L-HfNCl could be stable at or even above room temperature.

### Electronic structure and strain engineering

**Figure 3:**
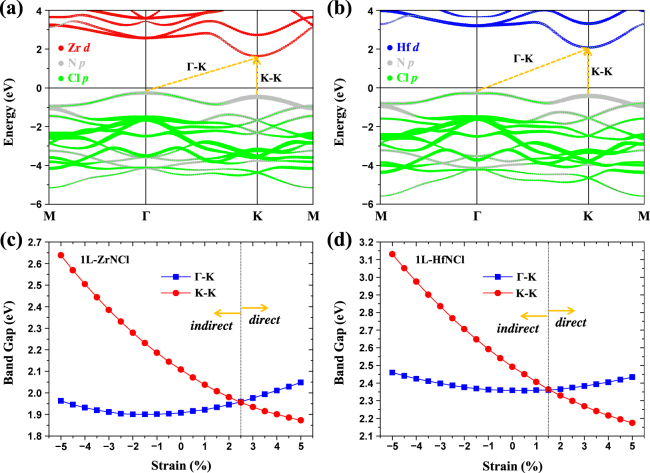
Electronic band structures of (**a**) 1L-ZrNCl and (**b**) 1L-HfNCl. Red, blue, gray, and green circles indicate the Zr *d*, Hf *d*, N *p*, and Cl *p* states, respectively, and the circle size is proportional to the weight of the state. Band gap as a function of the compressive or tensile strain with a range from −5% to 5% for (**c**) 1L-ZrNCl and (**d**) 1L-HfNCl. Here, the vertical dotted line indicates the strain at the indirect to direct band gap transition. Blue square and red circle symbols stand for Γ-K and K-K gaps, respectively.

Figure 3(a,b) show the orbital-decomposed electronic band structures of 1L-ZrNCl and 1L- HfNCl. Overall electronic band structures are similar to each other. For instance, at the equilibrium (unstrained) state, the electronic structures of both systems present an indirect gap incorporating the valence band maximum (VBM) at the Γ-point and the conduction band minimum (CBM) at the K-point. Quantitatively, the indirect band gaps of 1L-ZrNCl and 1L-HfNCl are estimated to be 1.91 eV and 2.36 eV, respectively. However, when we perform the calculation using the Heyd-Scuseria-Ernzerhof hybrid functional (HSE06)^35,36^, we find the indirect band gaps of 1L-ZrNCl and 1L-HfNCl to be 2.93 and 3.37 eV, respectively (Fig. S3). Except for the band gap size, we find that the band energy dispersions calculated by HSE06 and PBE functionals show qualitatively the same trends. For instance, both calculations indicate that 1L-ZrNCl or 1L-HfNCl is an indirect gap semiconductor from the Γ- to the K-point (Fig. S3). We also found that the valence band edge at the K-point (VBM at the Γ-point) is mostly from the N *p*_*x*/*y*_ orbitals (N *p*_*z*_ orbitals) and CBM at the K-point (the conduction band edge at the Γ-point) from the Zr ${d}_{xy/{x}^{2}-{y}^{2}}$ orbitals (Zr *d*_*xz*/*yz*_ orbitals). In addition, charge density distributions of VBM and CBM states for both 1L-ZrNCl and 1L-HfNCl are found to be around the N and Zr (or Hf) atoms, respectively (Fig. S4).

In order to investigate how the thickness (the number of layers) of ZrNCl and HfNCl affects electronic band structure, we performed band structure calculations for bulk and multi-layer (from 2L to 6L) ZrNCl and HfNCl. As a result, with an increase of the number of layers from 1L to bulk, the indirect band gaps of both ZrNCl and HfNCl monotonically decrease. Quantitatively, the band gaps of 1L-ZrNCl and 1L-HfNCl are larger than their bulks by 8.3 and 8.7%, respectively, while maintaining an indirect band gap, due to the quantum confinement effect (Fig. S5)^37^.

To gain physical insights into the strain-induced electronic properties, we investigated the strain effect on the band gap of 1L-ZrNCl and 1L-HfNCl. Figure 3(c,d) shows the strain dependence of the band gap of 1L-ZrNCl and 1L-HfNCl. Here, the strain *ε* can be defined as *ε* = (*a* − *a*_0_)/*a*_0_ = Δ*a*/*a*_0_, where *a* and *a*_0_ indicate the lattice constants of the strained and unstrained (equilibrium) systems, respectively. Note that the Γ-K and K-K gaps indicate the indirect and direct band gaps, respectively. It is noted that the slope of the K-K gap is sensitive compared to the Γ-K gap with respect to the compressive or tensile strain and the K-K gap is larger than the Γ-K gap at the equilibrium (unstrained case). The crossover between sizes of the two gaps, *i*.*e*., the indirect to direct band gap transition, occurs at the tensile strains of 2.5% and 1.5% for 1L-ZrNCl and 1L-HfNCl, respectively. After the transition, the (direct) band gap is found to decrease rapidly with respect to the strain. The strain-engineered direct band gap would make 1L-ZrNCl and 1L-HfNCl meaningful candidates for electronic and optoelectronic device applications.

### Electric transport properties

**Figure 4:**
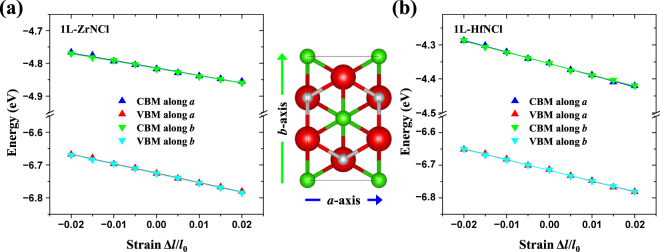
Shifts of the conduction and valence band edges measured from the vacuum level as a function of the uniaxial strains along *a*- and *b*-directions for (**a**) 1L-ZrNCl and (**b**) 1L-HfNCl. A rectangular supercell of 1L-ZrNCl is given between (**a**,**b**), which is double the size of the hexagonal primitive cell.

To investigate the electronic transport properties of 1L-ZrNCl and 1L-HfNCl, we calculated the carrier mobilities using the deformation potential (DP) theory. According to the theory, *i*.*e*., Eq. (1), we need three parameters, the DP constant *E*_1_, the carrier effective mass m*, and the 2D elastic modulus *C*_2D_. First, to estimate the DP constant, the shifts of the positions of CBM and VBM as a function of the uniaxial strain are plotted in Fig. 4. Moreover, to determine the shifts along the different carrier transport directions, *i*.*e*., *a*- and *b*-directions, we introduced a rectangular supercell shown in Fig. 4. However, for both systems, when the uniaxial strain is applied, the CBM and VBM decrease monotonically regardless of the direction. Consequently, the DP constants which can be obtained by the linear fitting of the data in Fig. 4 are not affected so much by the direction. Subsequently, the 2D elastic modulus is calculated by the quadratic fitting of the total energy as a function of strain. Finally, the effective masses are calculated from ${m}^{\ast }={\hslash }^{2}{[{\partial }^{2}\varepsilon (\overrightarrow{k})/\partial {k}^{2}]}^{-1}$ for highly symmetric points selected in the Brillouin zone. Thus, if the DP constant (*E*_1_), 2D elastic modulus (*C*_2D_), and effective mass (m*) are obtained, the carrier (electron or hole) mobilities are calculated from Eq. (1). All these data including the relaxation time (*τ* = *μ*_2*D*_ m*/*e*) are listed in Table 1.

**Table 1:** Deformation potential (DP) constant *E*_1_ (in eV), 2D elastic modulus *C*_2D_ (in Jm^−2^), effective mass m* (in *m*_*e*_), relaxation-time *τ* (in ps), and 2D carrier mobility *μ*_2D_ (in cm^2^ V^−1^ s^−1^) for electron (*e*) and hole (*h*) along *a*- and *b*-directions in 1L-ZrNCl and 1L-HfNCl at 300 K.

System	Carrier type	*E*_1_	*C*_2D_	m* (Γ-K-M)	m* (M-Γ-K)	*τ*	*μ*_2D_
1L-ZrNCl	*e* (*a*-axis)	−2.292	143.94	0.580	—	0.382	1.16 × 10^3^
	*h* (*a*-axis)	−2.878	143.94	—	−2.849	0.049	30.46
	*e* (*b*-axis)	−2.256	143.67	0.580	—	0.393	1.19 × 10^3^
	*h* (*b*-axis)	−2.883	143.67	—	−2.849	0.049	30.31
1L-HfNCl	*e* (*a*-axis)	−3.423	162.52	0.648	—	0.173	469.397
	*h* (*a*-axis)	−3.292	162.52	−1.268	10.217	0.012	2.04
	*e* (*b*-axis)	−3.324	162.08	0.648	—	0.183	498.38
	*h* (*b*-axis)	−3.244	162.08	−1.268	10.217	0.012	2.10

As listed in Table 1, we found that both the DP constant *E*_1_ and 2D elastic modulus *C*_2D_ show relatively weak direction- and carrier-type-dependences. In contrast, they reveal a strong asymmetry between electron and hole in the effective mass m*. For both 1L-ZrNCl and 1L-HfNCl, it should be noted that the effective mass of hole is found to be much larger than that of electron. This result can be well understood by the band curvature of CBM and VBM shown in Fig. 3. As a result, the obtained relaxation time strongly depends on the carrier type.

According to our calculation, for 1L-ZrNCl, the obtained electron mobility is 1.16 × 10^3^ cm^2^ V^−1^ s^−1^ (along *a*-direction) and 1.19 × 10^3^ cm^2^ V^−1^ s^−1^ (along *b*-direction), respectively. But the hole mobility remains to be just about 3% of the electron mobility, with a value of 30.46 cm^2^ V^−1^ s^−1^ (along *a*-direction) and 30.31 cm^2^ V^−1^ s^−1^ (along *b*-direction), respectively. This implies that the carrier mobility is highly asymmetric between electron and hole. The asymmetry is more strengthened in 1L-HfNCl. For 1L-HfNCl, the electron mobility along the *a*-direction (*b*-direction) is calculated to be 469.97 (498.38) cm^2^ V^−1^ s^−1^, whereas the hole mobility along the *a*-direction (*b*-direction) is 2.04 (2.10) cm^2^ V^−1^ s^−1^. Therefore, for both systems, the electric transport is electron dominated.

### Thermoelectric properties

The efficiency of the thermoelectric conversion can be evaluated by the dimensionless figure of merit, *ZT* = *S* ^2^ *σT*/*κ*, where *S*, *σ*, *T*, and *κ* are the Seebeck coefficient, the electrical conductivity, the absolute temperature, and the thermal conductivity, respectively. Hence, high *ZT* materials should have a high Seebeck coefficient or power factor defined as the product *S* ^2^ *σ*. Semiconducting TMDCs and layered materials have high Seebeck coefficient (which are in the range of 700–900 *μ*VK^−1^)^38,39^, indicating an appealing potential for thermoelectric applications^40,41^. Recently, unrivaled thermoelectric performance was discovered in bulk SnSe which is a layered orthorhombic structure^42^. After that, 1L-SnSe was predicted to show several times higher performance than the bulk SnSe^43^. These studies give us inspiration that high thermoelectric performance could be achieved in atomically thin 2D nanosheets.

**Figure 5:**
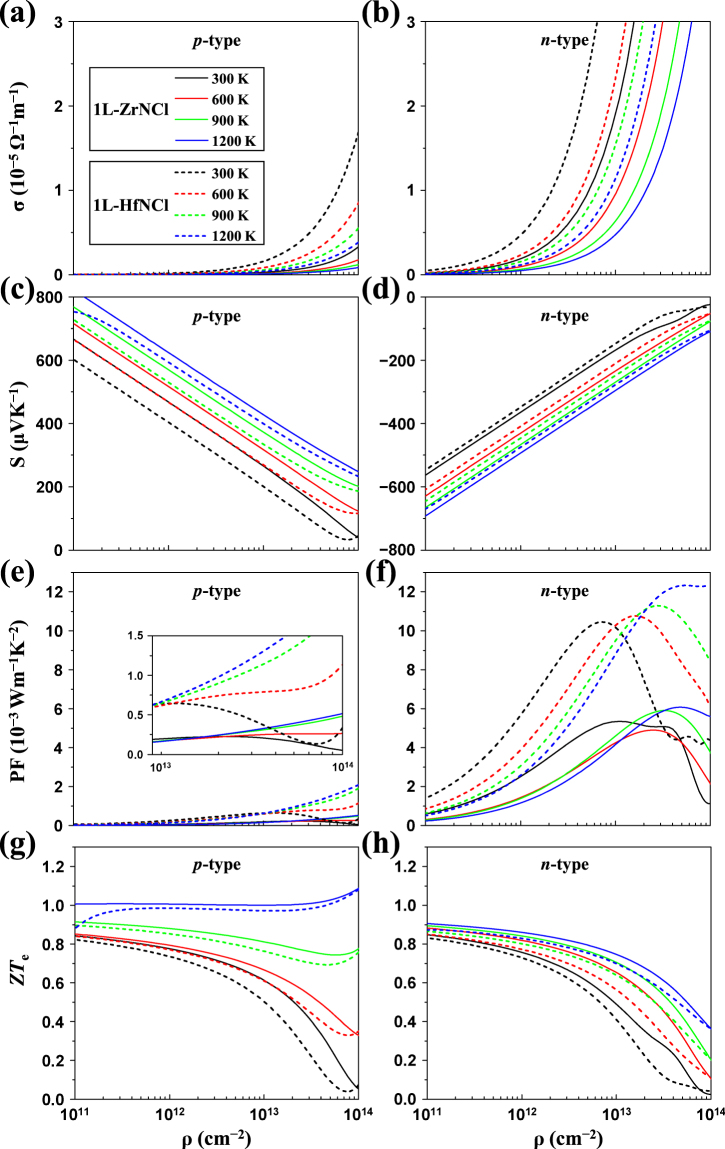
Transport coefficients of 1L-ZrNCl (solid lines) and 1L-HfNCl (dashed lines) as a function of the carrier density at 300, 600, 900, and 1200 K: (**a**,**b**) electrical conductivity *σ*, (**c**,**d**) Seebeck coefficient *S*, (**e**,**f**) power factor *S* ^2^ *σ*, (**g**,**h**) thermoelectric figure of merit (*ZT*_*e*_) from the electronic contribution. Note that left and right panels indicate *p*-type and *n*-type systems, respectively.

Figure 5 displays the electrical conductivity *σ* of the *p*- and *n*-type 1L-ZrNCl and 1L-HfNCl as a function of the carrier density *ρ* from 1 × 10^11^ cm^−2^ to 1 × 10^14^ cm^−2^ using the Boltzmann transport equation for electrons. Initially, the relaxation-time-divided electrical conductivity (*σ*/*τ*) is brought out by the BoltzTraP code. Because there are no available experimental data about the relaxation times of 1L-ZrNCl and 1L-HfNCl, we used the temperature-dependent relaxation time using the DP theory. For instance, to obtain the electrical conductivity of 1L-ZrNCl at 300 K, we adopted the averaged relaxation times at 300 K, i.e., 388 fs (for *n*-type) and 49 fs (for *p*-type) as listed in Table 1. Note that the relaxation time from the DP theory has no explicit carrier density dependence.

The electrical conductivity increases with respect to the carrier density, as shown in Fig. 5(a,b). Moreover, for both 1L-ZrNCl and 1L-HfNCl, we found that the electrical conductivity of the *n*-type system is significantly larger than the *p*-type system at the same carrier density, which is due to longer relaxation time of electrons compared to holes (see Table 1). In addition, at a fixed carrier density, the electrical conductivities of both the *p*-type and *n*-type 1L-ZrNCl and 1L-HfNCl decrease with the temperature. The reason is that, as the temperature rises, the scattering rate of carriers increases and the relaxation time decreases.

The Seebeck coefficient of a material is strongly dependent on the carrier density. According to Fig. 5(c,d), the Seebeck coefficients of the 1L-ZrNCl and 1L-HfNCl have values of 600–800 *μ*VK^−1^ at the carrier density ~10^11^ cm^−2^ for the *p*-type system and 600–700 *μ*VK^−1^ at the same density for the *n*-type system. It is noted that 1L-ZrNCl or 1L-HfNCl has relatively high Seebeck coefficients comparable to layered semiconducting TMDCs (700–900 *μ*VK^−1^)^38,39^. This finding implies that 1L-TMNHs would be good candidates for high-performance thermoelectric materials. A high power factor *S* ^2^ *σ* is another measure of high efficiency thermoelectric materials. In Fig. 5(e) of the *p*-type 1L-ZrNCl and 1L-HfNCl, the power factor is not affected too much by the temperature at the carrier density *ρ* < 10^13^ cm^−2^, whereas it rapidly increases with the temperature at the carrier density *ρ* > 10^13^ cm^−2^. Comparing between Fig. 5(e,f), it is found that the power factors of the *n*-type systems are considerably higher than those of the *p*-type ones, i.e., *n*-type power factors. This is reasonable because the *n*-type electrical conductivity is much larger than the *p*-type one. Finally, we present the thermoelectric figure of merit (*ZT*_*e*_) from the electronic contribution, which is given by *ZT*_*e*_ = *S* ^2^ *σT*/*κ*_*e*_. It should be noted that the formula ignores the lattice thermal conductivity (*κ*_*l*_) and *ZT*_*e*_ then corresponds to the maximum thermoelectric *ZT*. The calculated results are shown in Fig. 5(g,h), belonging to the range of 0.8–1.1 at low carrier density, which are comparable to those for IV–VI layered materials (GeS, GeSe, SnS, and SnSe) at 300 K^44^.

## Summary

In summary, we carried out the first-principles calculations to investigate the stability, electronic structure, electric transport, and thermoelectric properties of 1L-ZrNCl and 1L-HfNCl. The cleavage energies of both the ZrNCl and HfNCl systems were shown to be lower than that of graphite, signifying an easy cleavage. In addition, from the phonon dispersion spectrum and *ab initio* molecular dynamics simulation, those systems were shown to be thermodynamically stable. It was found that they undergo a transition from an indirect to direct band gap under the tensile strain and also reveal the electron-dominated electric transports with the maximum electron mobility of about 1.19 × 10^3^ cm^2^ V^−1^ s^−1^ (1L-ZrNCl) and 498 cm^2^ V^−1^ s^−1^ (1L-HfNCl), significantly higher than 1L-MoS_2_. Finally, thermoelectric properties were investigated to give relatively high Seebeck coefficients and high power factors. The easy cleavage, tunable band gap, high electron mobility, and high thermoelectric efficiency suggest that 1L-ZrNCl and 1L-HfNCl are novel promising candidates for application in nanoscale optoelectronic and thermoelectric devices.

## Electronic supplementary material

Supplementary Information
