# Supplementary material for: Two-dimensional semiconductors ZrNCl and HfNCl: Stability, electric transport, and thermoelectric properties
Source: Sci Rep. 2017 Dec 11;7:17330. doi: 10.1038/s41598-017-17590-w (PMC5725492; doi:10.1038/s41598-017-17590-w)
Supplement: Supplementary file 1 — Supplementary Information [file 41598_2017_17590_MOESM1_ESM.pdf]

Supplementary Information

Two-dimensional semiconductors ZrNCl and HfNCl: Stability, electric transport, and thermoelectric properties

Won Seok Yun and J. D. Lee*

Department of Emerging Materials Science, DGIST, Daegu 42988, Republic of Korea.
*Corresponding author: jdlee@dgist.ac.kr

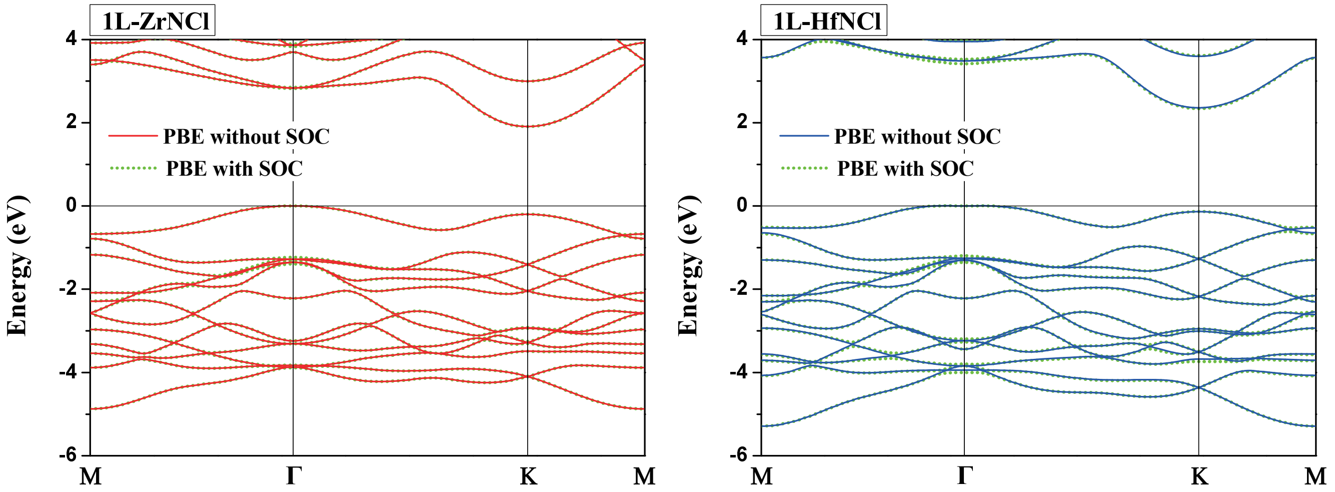

Figure S1. Electronic band structures of 1L-ZrNCl (left panel) and 1L-HfNCl (right panel) calculated by the PBE functional with spin-orbit coupling (SOC) (green dotted lines) and without SOC (red/blue solid lines). The valence band maximum is set to zero and inclusion of SOC does not change the electronic band structure.

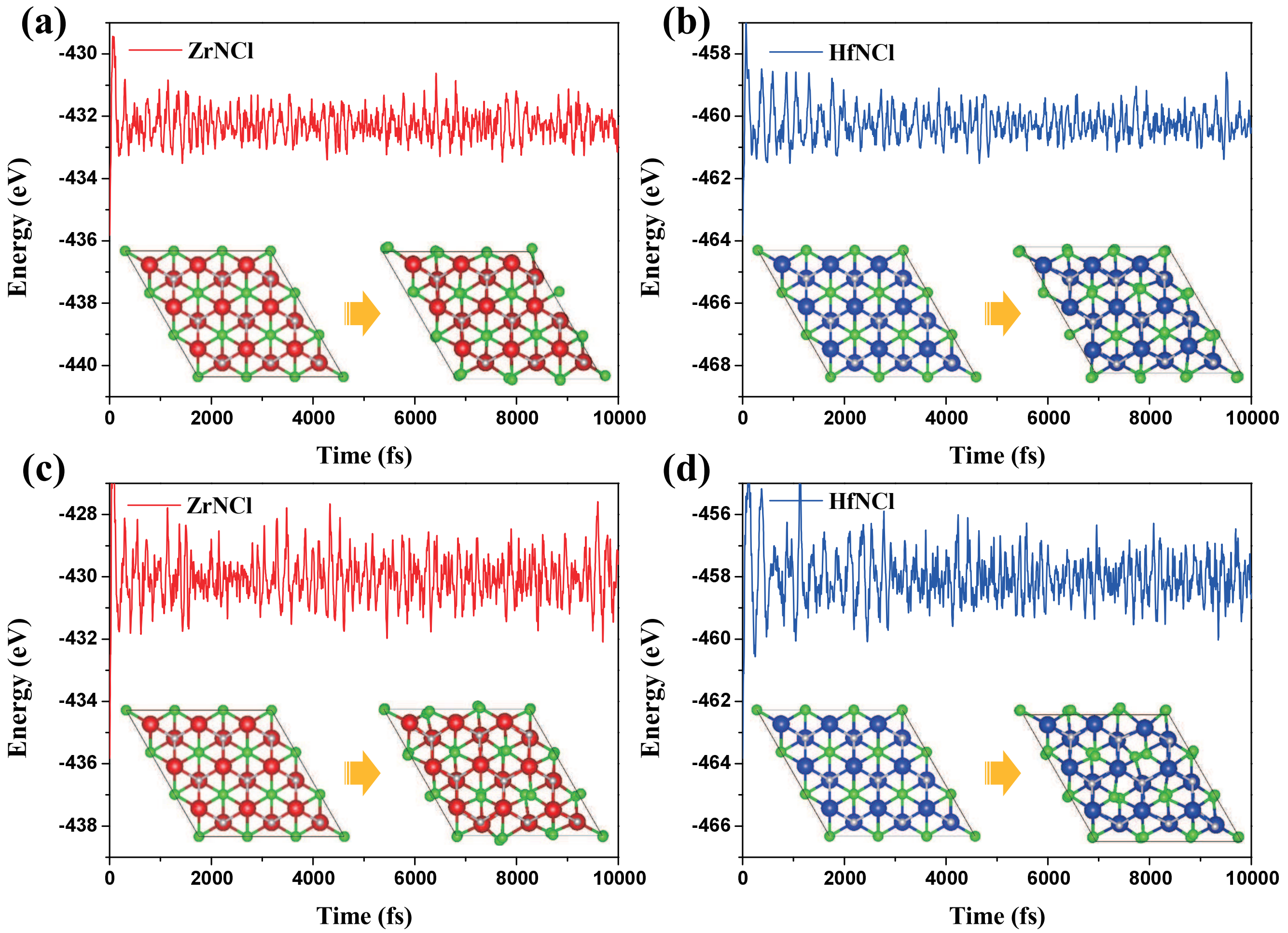

Figure S2. Total energy fluctuation during the AIMD simulation for 1L-ZrNCl (left panels) and 1L-HfNCl (right panels) at (a,b) 500 K and (c,d) 800 K. The insets in (a-d) show the snapshots of the atomic configuration at 0 ps (left) and 10 ps (right).

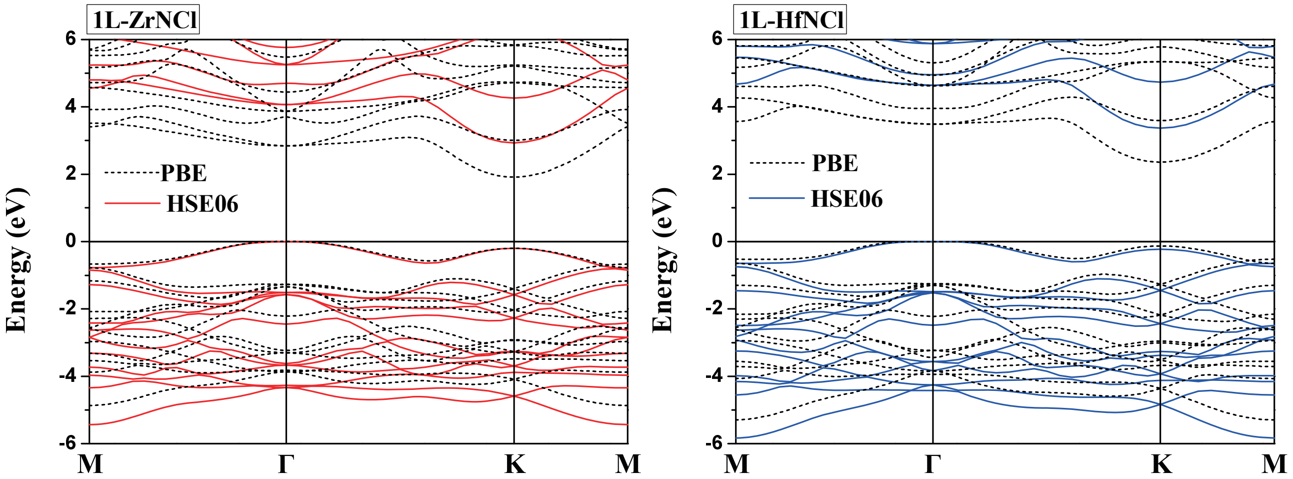

Figure S3. Electronic band structures of 1L-ZrNCl (left panel) and 1L-HfNCl (right panel) calculated with the PBE (dotted lines) and HSE06 (red/blue solid lines) functionals.

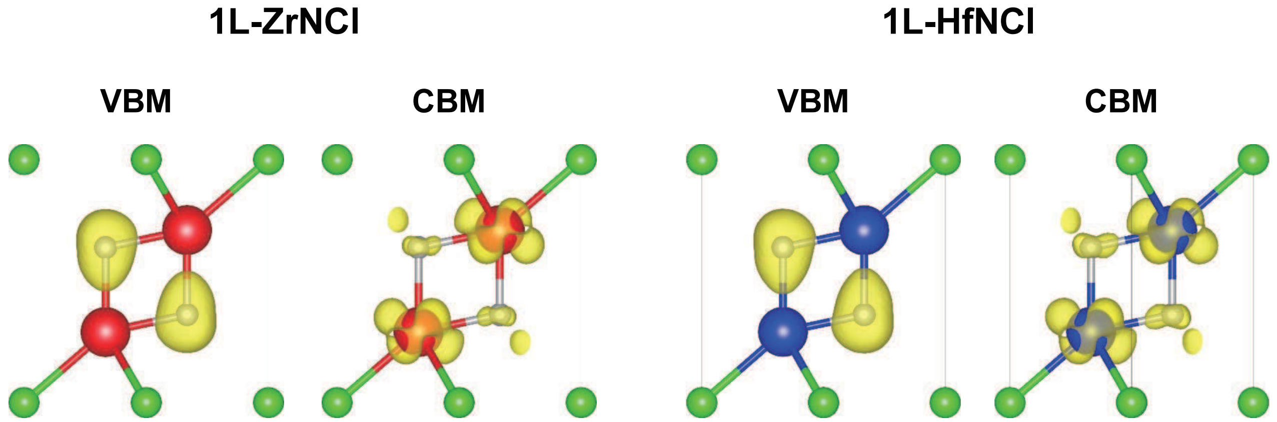

Figure S4. Side-views of 1L-ZrNCl (left panel) and 1L-HfNCl (right panel). The yellow distributions represent the charge density isosurfaces of the valence band maximum (VBM) and conduction band minimum (CBM) states. The isosurface value is taken as 0.01 $e\text{Å}^{-3}$.

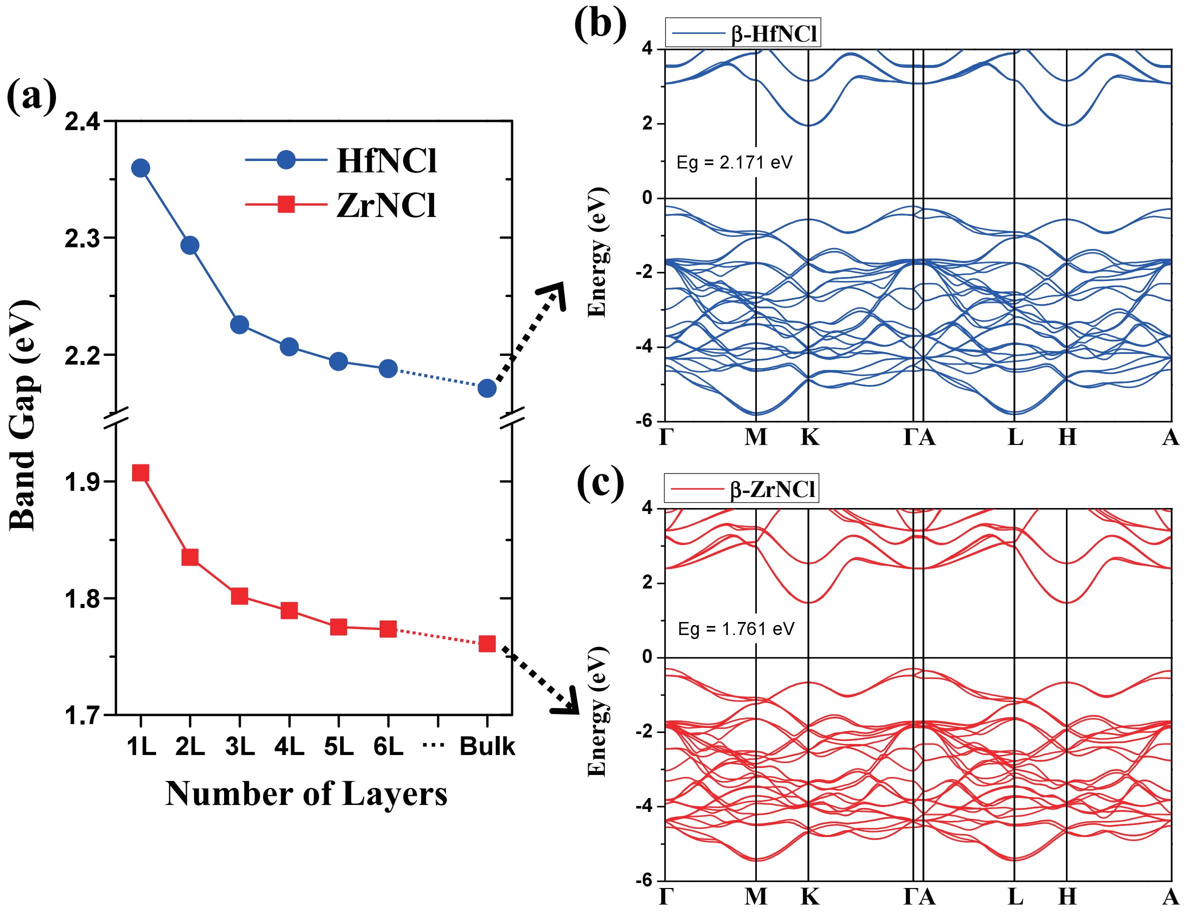

Figure S5. (a) Evolution of the indirect band gap (Γ-K) as a function of the thickness of both ZrNCl and HfNCl. Calculated electronic band structures of (b) bulk ZrNCl (lower panel) and (c) bulk HfNCl (upper panel).
